# Endoplasmic Reticulum Stress in Diabetic Nephrology: Regulation, Pathological Role, and Therapeutic Potential

**DOI:** 10.1155/2021/7277966

**Published:** 2021-08-02

**Authors:** Lihua Ni, Cheng Yuan, Xiaoyan Wu

**Affiliations:** ^1^Department of Nephrology, Zhongnan Hospital of Wuhan University, Wuhan 430071, China; ^2^Department of Gynecological Oncology, Zhongnan Hospital of Wuhan University, Wuhan 430071, China

## Abstract

Recent progress has been made in understanding the roles and mechanisms of endoplasmic reticulum (ER) stress in the development and pathogenesis of diabetic nephropathy (DN). Hyperglycemia induces ER stress and apoptosis in renal cells. The induction of ER stress can be cytoprotective or cytotoxic. Experimental treatment of animals with ER stress inhibitors alleviated renal damage. Considering these findings, the normalization of ER stress by pharmacological agents is a promising approach to prevent or arrest DN progression. The current article reviews the mechanisms, roles, and therapeutic aspects of these findings.

## 1. Introduction

Diabetic nephropathy (DN) is one of the common microvascular complications of diabetes. The main clinical manifestations are proteinuria, hyperglycemia, and impaired renal function. Additionally, mesangial hyperplasia, glomerular sclerosis, extracellular matrix accumulation, and tubulointerstitial fibrosis can be observed pathologically. Numerous studies have demonstrated the role of endoplasmic reticulum (ER) stress in the pathogenesis of DN [1–3].

The ER is an important subcellular organ in eukaryotic cells. It facilitates the synthesis and export of proteins and lipids [4]. It can fold proteins in the cisternae and transport synthesized proteins to the Golgi apparatus through vesicles. Several ER chaperone proteins, including protein disulfide isomerase, correct the folding of newly made proteins. Our article summarizes the role of ER stress in DN.

## 2. ER Stress

The ER is committed to protein folding, maturation, quality control, and trafficking. The ER becomes stressed (ER stress) because of the accumulation of newly synthesized unfolded proteins. Moderate ER stress promotes the stability and recovery of the intracellular environment. Persistent ER stress slows protein folding, contributing to the accumulation of misfolded and unfolded proteins. Thus, ER stress ultimately leads to apoptosis, protein degradation, translation attenuation, and an antioxidant response.

ER stress can be divided into three types: the unfolded protein response (UPR), the ER overload response (EOR), and sterol regulatory element-binding protein (SREBP). The UPR and EOR are attributed to disordered protein processing. SREBP results from the depletion of newly synthesized sterol in the ER.

### 2.1. UPR

The UPR is activated by the accumulation of unfolded proteins and increases protein folding capacity [5, 6]. The UPR is regulated by three sensors to restore the normal function of the ER: protein-kinase-RNA-like ER kinase (PERK), inositol-requiring enzyme 1 (IRE-1), and activating transcription factor 6 (ATF6).

#### 2.1.1. PERK

PERK is a transmembrane protein in the ER [7, 8]. Under normal conditions, PERK is inactivated by binding to glucose-regulated protein 78 (GRP78). Under pathological circumstances (such as hypoxia, ischemia, and oxidative stress), PERK is activated as a homodimer by dissociating from GRP78. Phosphorylated PERK phosphorylates eukaryotic translation initiation factor (eIF2), which attenuates mRNA translation. Additionally, phosphorylated PERK induces the transcription factors ATF4 and C/EBP homologous protein (CHOP), which regulate the expression of genes involved in maintaining homeostasis [9]. Activated PERK also increases the expression of the transcription factor NRF2, which triggers an antioxidant response [10, 11].

#### 2.1.2. IRE-1

IRE-1 is highly conserved in the ER. Interestingly, this protein is a kinase and an endoribonuclease. The RNase activity of IRE-1 increases the degradation of RNA and subsequently reduces protein synthesis. Previously, IRE-1 was shown to regulate cell survival and apoptosis [12]. Recent studies have changed this concept by demonstrating that IRE-1 directs UPR signaling and cell fate [13]. X-box binding protein 1 (Xbp-1) was identified as a target of IRE-1 [14]. To enhance protein folding abilities, IRE-1 induces the mRNA expression of Xbp-1 and subsequently augments the transcription of ER quality-control components.

It has been proposed that IRE-1 can regulate apoptosis during ER stress. Repression of IRE-1 potentiates cell apoptosis by upregulating caspase-2 (Casp2). Additionally, activation of IRE-1-Casp2 events triggers cell death in the apoptotic phase.

#### 2.1.3. ATF6

As the third sensor of the UPR, ATF6 is a transmembrane protein and transcription factor. Under conditions of non-ER stress, ATF6 is constitutively located in the ER and bound to GRP78 [15]. Under conditions of ER stress, ATF6 can translocate to the Golgi, where it is cleaved following GRP78 dissociation. Cleaved ATF6 can translocate to the nucleus and trigger Xbp-1 gene expression, providing an additional substrate for IRE-1 [16, 17]. Some UPR proteins (such as ER chaperones) are direct targets of cleaved ATF6, which enhances ER protein folding capacity.

In summary, the UPR has evolved into an interconnected, dynamic, and flexible network of tubular and planar membranes. Under conditions of ER stress, the UPR attenuates the unfolded protein load in the ER. If successful in decreasing the number of misfolded proteins, the UPR is alleviated, and the cell survives. If ER stress persists and cannot be restored, the UPR induces apoptosis. Significantly, IRE-1 is necessary and sufficient to induce apoptosis, while PERK and AFT6 are dispensable in apoptosis. In certain cases, different sensors of ER stress might act as major executioners of cell death.

### 2.2. EOR

In contrast to the UPR, the EOR is characterized by the release of Ca^2+^ from the ER lumen to trigger the expression of reactive oxygen species (ROS) [18, 19]. The enhanced ROS production activates NF-*κ*B, namely, the EOR-Ca^2+^-ROS-NF-*κ*B pathway. NF-*κ*B is a transcription factor that regulates the expression of many genes involved in cell survival and cell proliferation. Interestingly, the EOR-Ca^2+^-ROS axis regulates cell death associated with oxidative stress and apoptosis, while the EOR-Ca^2+^-ROS-NF-*κ*B axis mediates cell viability due to the effects of NF-*κ*B. The downstream target of NF-*κ*B has antiapoptotic effects. Thus, ROS-induced NF-*κ*B alleviates the detrimental effects of ROS.

In summary, the EOR-Ca^2+^-ROS pathway has two opposing effects on cell viability: the EOR-Ca^2+^-ROS pathway enhances apoptosis, while the ROS pathway activates NF-*κ*B and promotes cell viability [20].

### 2.3. SREBP

Intracellular cholesterol depletion leads to ER stress-induced SREBP activation [21]. Several publications have also demonstrated that ER stress is related to activated SREBP and lipid disorders [22–24]. Mammalian cells express three SREBP isoforms: SREBP-1a, SREBP-1c, and SREBP-2. SREBP-1a and SREBP-1c were reported to be associated with the biosynthesis of cholesterol and fatty acids. SREBP-2 is a more selective activator of cholesterol biosynthesis.

Current studies suggest three potential mechanisms of ER stress-induced activation of SREBP [25–27]: caspase-induced SREBP cleavage, eIF2 phosphorylation-dependent downregulation of insulin-induced gene-1 and 2 (INSIG), and GRP78 dissociation from the SREBP cleavage-activation protein (SCAP)-SREBP complex.

Overall, ER stress is the first attempt to regulate protein folding demands, ER overload, and intracellular cholesterol depletion to restore homeostasis and functions to keep the cell alive and restore cellular functions. Each ER stress signal has unique and distinct targets, which act as homeostatic feedback loops to control ER stress [28]. If successful in restoring homeostasis, ER stress is alleviated, and the cell survives. However, if adaptive responses are lost, ER stress continues, leading to high or chronic ER stress. Unalleviated ER stress promotes cell death.

## 3. Mechanism of ER Stress in DN

DN is the leading cause of morbidity and end-stage renal disease. Hyperglycemia induces ER stress and apoptosis in renal cells [29]. Accumulating evidence has demonstrated that ER stress plays a substantial role in the development and pathogenesis of DN. The exact mechanisms are complex, have not been fully elucidated, and are summarized as follows:

### 3.1. Renal Epidermal Growth Factor Receptors (EGFRs)

Previous studies have reported that EGFRs are activated in DN. Zhang et al. [30] demonstrated that inhibiting renal EGFRs with erlotinib reduced kidney ER stress and alleviated nephropathic changes in STZ mice, a finding likely associated with the inhibition of mTOR and activation of the AMPK pathway. Xu et al. [31] suggested that EGFR plays an essential role in the development of DN by enhancing ROS production and ER stress. Inhibiting EGFR alleviated renal damage via the EGFR/AKT/ROS/ER stress signaling pathway. Thus, direct inhibition of EGFR activity and/or inhibition of signaling pathways activated by EGFRs might be novel strategies to prevent and treat progressive renal damage in DN.

### 3.2. Hyperglycemia

Absolute or relative insulin deficiency leads to elevated blood glucose levels, termed hyperglycemia. Chronic high glucose (HG) exposure activates IRE1 to splice Xbp-1 mRNA, whereas acute exposure to HG activates IRE without Xbp-1 splicing [32]. Additionally, the activation of UPR pathways is amplified by supplementation with HG and FFAs [33]. HG stimulates renal proximal tubular cells and increases the accumulation of ROS, leading to enhanced ER stress. Several studies have shown HG-induced ER stress in vivo and in vitro [34, 35]. Improving glucose metabolism might highlight one of the molecular mechanisms for clinical DN treatment in the future.

### 3.3. Reactive Oxygen Species

ROS play dual roles in ER stress [36]. On the one hand, ROS act as signaling intermediates that report ER stress to the UPR. Consequently, ER stress can be mitigated. On the other hand, when ER stress is not relieved over time, the delayed expression of proteins such as CHOP initiates a secondary increase in ROS. Additionally, the induction of ER oxidase 1 and calcium transfer across specialized ER-mitochondria leads to a secondary increase in ROS, contributing to cell death. The complexities of how ROS are formed and contribute to both homeostatic signaling and cell death raise numerous challenges in translating recent findings into clinical applications.

### 3.4. Angiotensin II Receptor Pathway

It is widely accepted that activation of the renin-angiotensin system (RAS), particularly the intrarenal RAS, plays a significant role in the pathophysiology of DN. Angiotensin II (Ang II), a profibrotic and proinflammatory peptide, is the major factor of the RAS. Angiotensin II (Ang II) plays a negative role in ER stress-induced apoptosis. Sun et al. [37] suggested that angiotensin-converting enzyme inhibitors (ACEIs) decrease ER stress-induced renal apoptosis in animal models of diabetes. Additionally, Ang II is associated with increased expression of ER chaperones and GRP78 (an ER stress marker) [38].

### 3.5. Free Fatty Acids (FFAs)

Dietary fats and changes in lipid metabolism due to diabetes could contribute to increased FFA concentrations [39]. FFAs play a significant role in the pathogenesis of DN [40, 41]. Saturated FFAs such as palmitic acid are proapoptotic factors in *β*-cells [42], while monounsaturated FFAs such as palmitoleic and oleic acid are capable of preventing/alleviating palmitic acid-induced apoptosis in pancreatic *β*-cells [43]. Palmitic acid induces ER stress in podocytes, leading to an unfolded protein response. Additionally, Sieber et al. [44] showed that monounsaturated palmitoleic acid and oleic acid decrease palmitic acid-induced cell death. Dietary shifting of the FFA balance toward unsaturated FFAs can delay the progression of DN.

### 3.6. Advanced Glycation End Products (AGEs)

The formation of AGEs is associated with hyperglycemia. Emerging studies [45, 46] have indicated that the accumulation of AGEs induces podocyte apoptosis through ER stress, leading to albuminuria and renal damage [44, 47]. Additionally, AGEs induce enhanced GRP78 expression [48], which can be alleviated by TUDCA (an ER stress inhibitor mentioned previously) [49]. Thus, AGEs trigger ER stress in DN.

### 3.7. X-Box Binding Protein 1 (Xbp-1)

Xbp-1 is a downstream transcription factor stimulated by ER stress that is spliced by activated IRE1*α* [50, 51]. Increased spliced Xbp-1 (sXbp-1) reverses HG-induced reactive oxygen species production and extracellular matrix expression [29]. However, knocking down intrinsic sXbp-1 expression induces opposite effects. These findings suggest that the sXbp-1 pathway in ER stress plays a significant role in HG-induced oxidative stress and extracellular matrix synthesis [52].

### 3.8. Autophagy

Autophagy plays multiple roles in cells, such as those in cell growth, differentiation, and death [53]. The interaction of ER stress and autophagy in DN has been studied [54–56]. The basal level of autophagy in podocytes was reduced in an animal model of streptozotocin-induced diabetes. Similarly, the levels of autophagy markers were decreased in cultured glomerular epithelial cells (GECs) exposed to HG. The permeability of GECs was damaged by HG levels and alleviated by stimulating autophagy using rapamycin. Interestingly, TUDCA and salubrinal (both of which are ER stress inhibitors) attenuated HG-induced autophagy suppression.

## 4. Roles of ER Stress in Various Renal Cells in DN

Recent studies have demonstrated that ER stress is closely related to podocyte injury, glomerular endothelial cells (GECs), mesangial cells (MCs), and tubular epithelial cells. In patients with DN, hyperglycemia can induce ER stress in various ways, leading to cellular injury.

### 4.1. Podocytes

Podocyte injury is vital in the progression of DN. Emerging evidence suggests that ER stress is stimulated in patients with DN. Podocytes are likely susceptible to ER stress because of their large ER capacity and high levels of anabolic or catabolic activities.

AGEs and HG can trigger ER stress and apoptosis in podocytes that can be alleviated by ER stress suppressors. Tian et al. [57] suggested that emodin ameliorates renal damage in DN mice. Emodin decreases HG-induced ER stress in podocytes by counteracting the upregulation of phosphorylated PERK, phosphorylated eIF2*α*, ATF4, and CHOP [57]. Sieber et al. [44] showed that palmitic acid induces ER stress in podocytes and that palmitoleic acid and oleic acid alleviate the palmitic acid-induced UPR and prevent podocyte death. Yu et al. [58] suggested that Ang II induces podocyte foot process fusion and apoptosis via ER stress, effects that can be attenuated by curcumin.

Fan et al. [59] demonstrated that reticulon 1A (RTN1A) might be a key regulator of ER stress in podocytes in DN. In db/db mice with unilateral nephrectomy (an animal model of progressive DN), RTN1A expression and ER stress were increased. In cultured podocytes, RTN1A mediates albumin-induced ER stress and apoptosis. The positive feedback loop between RTN1A and CHOP contributes to enhanced ER stress in podocytes.

Crosstalk between mTOR signaling and ER stress in podocytes has emerged recently. Lei et al. [60] demonstrated that HG induces podocyte injury through activated mTOR-induced ER stress and apoptosis.

Collectively, ER-induced podocyte injury is crucial for DN. Several stimulators (such as HG, AGEs, FFAs, and Ang II) induce ER stress in podocytes. RTN1A and mTOR might participate in these processes. Sustained ER stress leads to podocyte apoptosis and ultimately cell death. Indeed, the stimulators and mechanisms are largely unclear and require further study.

### 4.2. MCs

MCs are the principal components of the glomerular mesangium. MC injury leads to renal dysfunction, contributing to DN. Previous studies have demonstrated that ER stress induces MC injury in DN, which can be summarized as follows:

Hyperglycemia and ROS are widely recognized to stimulate ER stress in cultured MCs. Yao et al. [61] investigated the mechanism of HG-induced ER stress in MCs and found that HG induces ER stress through fatty acid-binding protein 4 (FABP4), a carrier protein for fatty acids. Hyperglycemia increases ROS in DN. Previous studies have demonstrated that increased ROS induce ER stress, leading to cell apoptosis. Xu et al. [31] suggested that the EGFR-AKT-ROS-ER stress pathway is present in STZ-induced diabetic mice and HG-treated MCs, and blocking EGFR might be a therapeutic strategy for DN.

The expression of profibrotic transforming growth factor-*β*1 (TGF-*β*1) is increased in MCs in diabetes. Several factors (such as And II and HG) associated with DN can increase the expression of TGF-*β*1 in MCs in vivo and in vitro [62–66]. Xu et al. [67] found that TGF-*β*1 induces ER stress in MCs, an effect that can be augmented by E-prostanoid 2 receptor (EP2) deficiency.

Lipid-mediated ER stress in MCs was also studied. Park et al. [68] cultured rat MCs with palmitate, which mimics lipotoxicity, to determine the mechanism of lipotoxicity-induced mesangial cell injury and pathogenesis of DN. Researchers found that lipotoxicity induced ER stress via the protein arginine methyltransferase 1 (PRMT1), which exacerbated MC apoptosis. Interestingly, Yang et al. [69] proved that inflammation accelerates lipid-induced MC injury through ER stress. Hence, strategies to mediate the expression of PRMT1 can be applied to prevent or limit DN.

Asymmetric dimethylarginine- (ADMA-) induced ER stress in DN should be mentioned. Increased renal ADMA levels have been reported in DN [70]. Park et al. [71] demonstrated that elevated ADMA levels induce ER stress, leading to mitochondrial membrane potential injury and apoptosis in MCs.

In summary, hyperglycemia, ROS, TGF-*β*1, and ADMA can induce ER stress in MCs. Sustained ER stress leads to MC apoptosis, which contributes to renal damage in DN.

### 4.3. ECs

Accumulating evidence supports that glomerular endothelial cells (GEnCs) are principally involved in the process of DN. However, the relationship between ER stress and GEnCs remains unclear.

Bi et al. [72] suggested that Ang II induces ER stress, leading to GEnC injury and accelerated renal damage. Angiopoietin 1 (Angpt1), a member of the angiopoietin family of growth factors, could attenuate these changes through the Tie2 receptor-ERK1/2-p38 MAPK pathways.

As mentioned previously, the serum concentration of ADMA increases early, even when the glomerular filtration rate (GFR) is still within the normal range in chronic kidney disease [73]. Guo et al. [74] suggested that ADMA promotes ER stress and GEnC apoptosis. Quercetin, one of the most important flavonoids, has been shown to attenuate ADMA-induced ER stress in GEnCs.

Advanced oxidation protein products (AOPPs) have been indicated to contribute to the development of DN. Liang et al. [75] pointed out that AOPPs induced ER stress in GEnCs by increasing the expression of GRP78 and CHOP, contributing to endothelial-to-mesenchymal transition.

To date, only a few reports have focused on the roles of ER stress in GEnCs in DN. Further studies are needed to improve the understanding of the exact mechanism of ER stress in GEnCs.

### 4.4. Tubular Epithelial Cells

Renal tubular epithelial cells (RTECs) are the key target cells that are highly vulnerable to damage in the context of diabetes and play a significant role in the development of DN. ER stress in RTECs is considered to contribute to renal injury in DN.

Previous studies have proven that HG and albumin induce ER stress in TECs. Shibusawa et al. [76] observed that HG induces ER stress through the eIF2*α*-ATF4-CHOP pathway in HK2 cells (a proximal tubular cell line). Dapagliflozin attenuates HG-induced ER stress in vivo and in vitro. Sun et al. used the rat RTEC line NRK-52E to investigate HG-induced ER stress, revealing that the histone deacetylase inhibitor valproic acid alleviates HG-induced ER stress. Jia et al. [77] examined albumin-induced ER stress in HK2 cells and found that albumin stimulated miR-4756 expression in HK2 cells. Furthermore, overexpression of miR-4756 enhanced albumin-induced ER stress by targeting Sestrin2 in HK2 cells. These observations were further supported by Lindenmeyer et al. [29], who investigated proteinuria- and hyperglycemia-induced ER stress in renal biopsies from patients with DN and cultured renal epithelial cells. The researchers pointed out that HG and albumin induced ER stress by producing free radicals, aberrant protein glycosylation, or increased membrane and protein turnover.

Urotensin II (UII) and its receptor are highly expressed in renal tissue in patients with DN [78]. Pang et al. [79] observed increased UII expression and ER stress in DN patients and diabetic mice, and UII induced epithelial-to-mesenchymal transition (EMT) via the ER stress pathway in cultured HK2 cells. Additionally, a UII receptor antagonist and 4-PBA inhibited UII-induced ER stress and EMT.

PRMT1, which induces ER stress in the MCs, is upregulated in the serum of DN patients and triggers ER stress by activating PERK and ATF6 in HK2 cells [34]. Additionally, receptors or AGEs regulate ER stress in RTECs, which induce premature senescence through p21 signaling in DN [46]. Palmitic acid (PA) was also observed to activate ER stress in proximal tubular cells [80].

Overall, ER stress-induced tubular injury is significant in DN.

## 5. Targeting ER Stress in DN

Substantial progress has been achieved in treatments that target ER stress in DN. Food and Drug Administration- (FDA-) approved chemical chaperones, such as 4-PBA and TUDCA, are classic ER stress suppressors that enhance ER folding capacity, restore glucose tolerance, and improve insulin sensitivity in metabolic disorders associated with diabetes.

Several studies have demonstrated the therapeutic effects of herbal or plant medicines in the treatment of DN through targeting ER stress.

1. Astragaloside IV: astragaloside IV (AS-IV) is a saponin and active component in *Astragalus membranaceus* Bge, and AS-IV exerts protective effects against renal ER stress in vivo and in vitro [81, 82]. Subsequently, Guo et al. [83] found that AS-IV alleviates ER stress-induced podocyte injury by upregulating sarco-ER Ca^2+^-ATPase 2 (SERCA2) expression in STZ-induced DN rats
2. Huangkui: Huangkui is a traditional Chinese medicine that was approved by the China State FDA (z19990040) to treat nephritis. Ge et al. [84] demonstrated that Huangkui could improve renal damage by alleviating ER stress in DN rats
3. Oleanolic acid (OA): OA naturally occurs in fruits and vegetables. Several studies [85, 86] have reported that OA has antioxidant, antiglycation, anti-inflammatory, and microbicidal activities. Additionally, OA has therapeutic effects on DN via ER stress reduction [87]
4. Quercetin: quercetin is a potent antioxidant, and its function has been extensively studied because of its widespread distribution in various foods. Previous studies have demonstrated that quercetin exerts pharmacological effects, such as inhibiting platelet aggregation, hypertension, and lipid peroxidation [88–91]. In particular, in recent years, quercetin was found to improve diabetes-induced endothelial dysfunction by suppressing the ER stress pathway [74, 92]
5. Arctigenin: arctigenin (ATG) is a natural lignan compound mainly derived from the seeds of *Arctium lappa*. ATG has several bioactivities, such as anti-inflammatory, antioxidative, and antidiabetic activities. Additionally, ATG has been demonstrated to be an ER stress inhibitor that suppresses the renal UPR in diabetic db/db mice and cultured HK2 cells [93]

Additionally, several Western medicines can inhibit ER stress in DN.

1. Aliskiren and valsartan: overactivation of the RAS plays significant roles in the progression of DN. Drugs targeting the RAS, including angiotensin-converting enzyme inhibitors (ACEIs) and Ang II type I receptor blockers (ARBs), have been used to cure DN. Aliskiren (an ACEI) and valsartan (an ARB) inhibit ER stress. Dual treatment with aliskiren and valsartan induces additive therapeutic effects in the treatment of DN [94]
2. Cannabinoid receptor 1 antagonist: cannabinoid receptor 1 (CB_1_R) is highly expressed in the kidneys of diabetic mice. CB_1_ inhibition prevents diabetes-induced renal damage. CB1 mediates HG- [95] or palmitic acid-induced ER stress/apoptosis [96] in cultured rat MCs and human RTECs
3. Chemical chaperones: chemical chaperones are small molecules that stabilize protein conformation, improve the ER folding capacity, and facilitate the trafficking of mutant proteins. These compounds include tauroursodeoxycholic acid (TUDCA) and 4-phenylbutyrate (4-PBA)

TUDCA is a secondary bile acid [97] that has been proven to have beneficial effects on various diseases, such as diabetes, obesity, and neurodegenerative diseases. The mechanism of these cytoprotective activities is largely due to the alleviation of ER stress.

4-PBA is a low-molecular-weight chemical chaperone that is widely used for urea cycle disorders. It has been demonstrated that 4-PBA can suppress ER stress, leading to the normalization of hyperglycemia and insulin resistance [98–100]. Cao et al. [101] suggested that 4-PBA prevents ER stress-induced podocyte apoptosis in type 2 diabetic mice. Additionally, three widely used ER stress inducers, tunicamycin, DTT, and MG132, were applied to evaluate ER stress in animals and cell lines. 4-PBA rescued drug-induced ER stress [102].

Additionally, some agents targeting pathways that exhibit remarkable functions can modify ER stress. GSK2606414 (inhibition of PERK), MKC-3946 (inhibition of IRE1*α*), salubrinal (inhibition of eIF2*α* phosphatases), and trazodone (inhibition of ATF4 induction) are widely used to attenuate ER stress in vivo and in vitro.

Finally, some old drugs exert novel effects in inhibiting ER stress in DN and were used only in research phases. Emodin, EGFR inhibitors, and dapagliflozin have been found to exert potential effects on ER stress and improve renal function in vitro and in animal studies. Furthermore, Shih et al. found that dapagliflozin treatment suppresses myocardial ER stress in patients with DN [103].

In summary, attention should be given to the treatment of ER stress. First, the abovementioned drugs may have multiple biological effects, indicating that these drugs are not specific in suppressing ER stress. Hence, the development of new medications that target ER stress is urgently needed. Additionally, the ER stress response can be a double-edged sword. Moderate ER stress restores the intracellular environment, while sustained ER stress contributes to renal damage in DN. Focused studies on ER stress in humans are encouraged [104–106]. Thus, a better understanding of the mechanism of this process would be helpful to improve therapies for DN.

## 6. Conclusion and Perspectives

**Figure 1:**
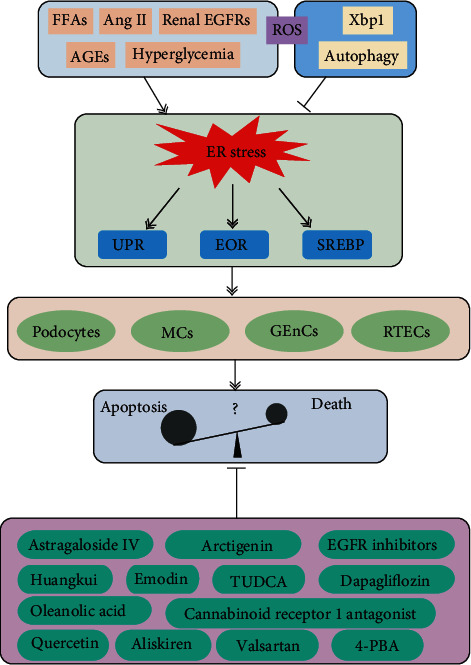
Role of endoplasmic reticulum (ER) stress in diabetic nephropathy. In diabetes mellitus, various effectors, including FFAs, Ang II, renal EGFRs, AGEs, and hyperglycemia, contribute to ER stress. Xbp-1 and autophagy can inhibit ER stress. The ROS had dual effects on ER stress. ER stress can be divided into three types: the UPR, the EOR, and SREBP. ER stress is closely related to podocyte injury, glomerular endothelial cells (GECs), mesangial cells (MCs), and renal tubular epithelial cells (RTECs). Several drugs have been demonstrated to inhibit ER stress in DN.

ER stress can be induced by several stimuli that alter homeostatic cellular functions (Figure 1). Increasing evidence suggests that ER stress plays important roles in the development and progression of DN.

Our understanding of the mechanism of acute and chronic ER stress in DN is rapidly increasing. ER stress clearly has both useful and harmful effects on the kidney. ER stress can recover normal organ function. However, chronic activation of ER stress can lead to chronic renal damage, which is associated with chronic renal failure. The upregulation of ER stress (by one or two ER stress markers) should not be interpreted as harmful. Additionally, some questions should also be resolved: Does activation of one pathway constitute the ER stress response, or must all three sensors (PERK, IRE-1, and ATF6) of the UPR be activated to be considered a true ER stress response? Do specific inducers of the UPR activate all three arms of the UPR?

**Figure 2:**
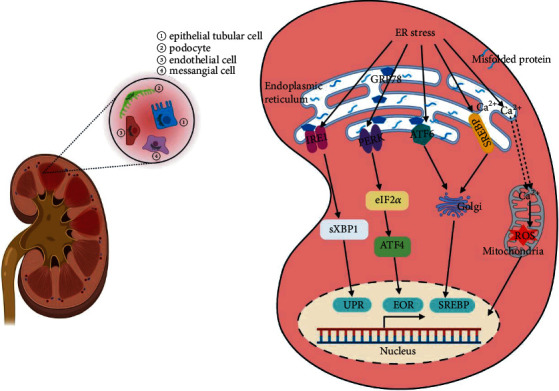
Endoplasmic reticulum (ER) stress in various renal cells in diabetic nephropathy (DN). ER stress participates in the development and pathogenesis of DN. When the ER is stressed, for example, by misfolded proteins or impaired Ca^2+^ homeostasis, the UPR, EOR, or SREBP is initiated. ER stress is mainly located in podocytes, glomerular endothelial cells, mesangial cells, and renal tubular epithelial cells in DN.

In this review, we summarized the roles of ER stress in different kidney cell types in DN (Figure 2). Many questions persist, mainly concerning which renal cells are the most affected by ER stress and whether all cells are affected similarly. These questions deserve further research. Additionally, evaluation of the temporal patterns of ER stress activation was neglected. Timing seems to play an important role in outcomes.

An improved understanding of the mechanisms of ER stress will lead to improved therapeutic strategies targeting ER stress as a treatment for DN. Considering the protective effects of ER stress, the blind pursuit of ER stress inhibition might undoubtedly be a mistake. Considering the adverse effects of ER stress, the development of new medications that can target ER stress in a cell- and disease-specific manner is urgently needed. Therapies targeting ER stress have shown increased potential and bright prospects. Focused studies on ER stress in humans would increase our understanding of targeted DN therapies.

In summary, ongoing research is required to solve these problems. Finally, the development and knowledge gained would similarly help to promote the treatment of both DN and ER stress-related disorders.

## Data Availability

The data used to support the findings of this study are available from the corresponding author upon request.
